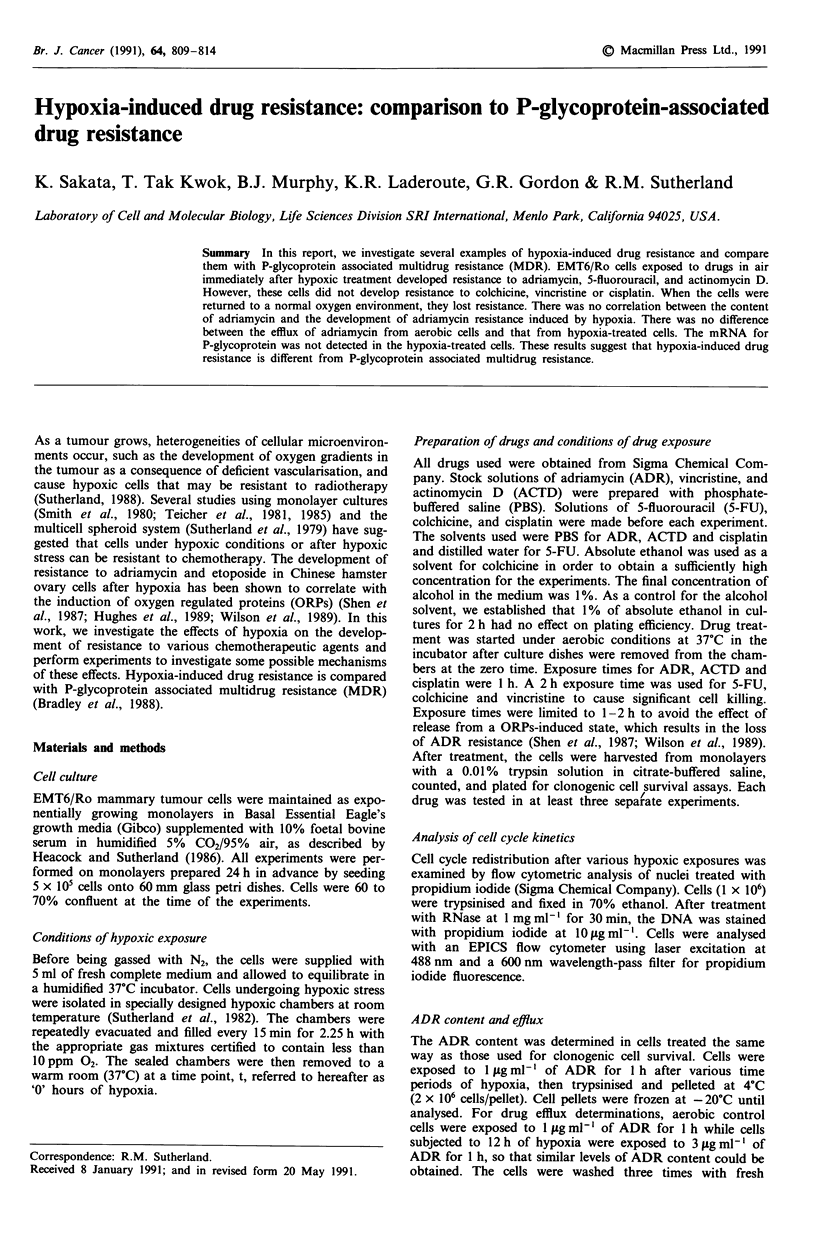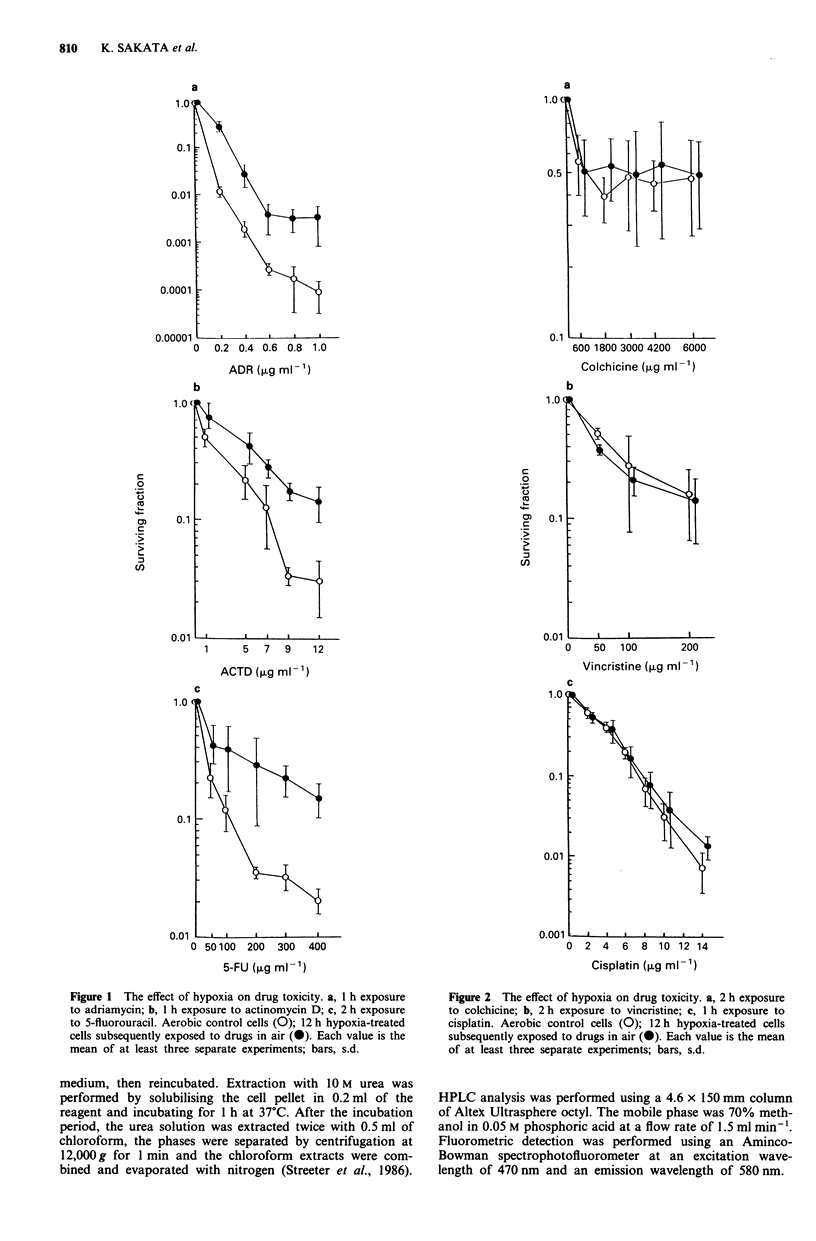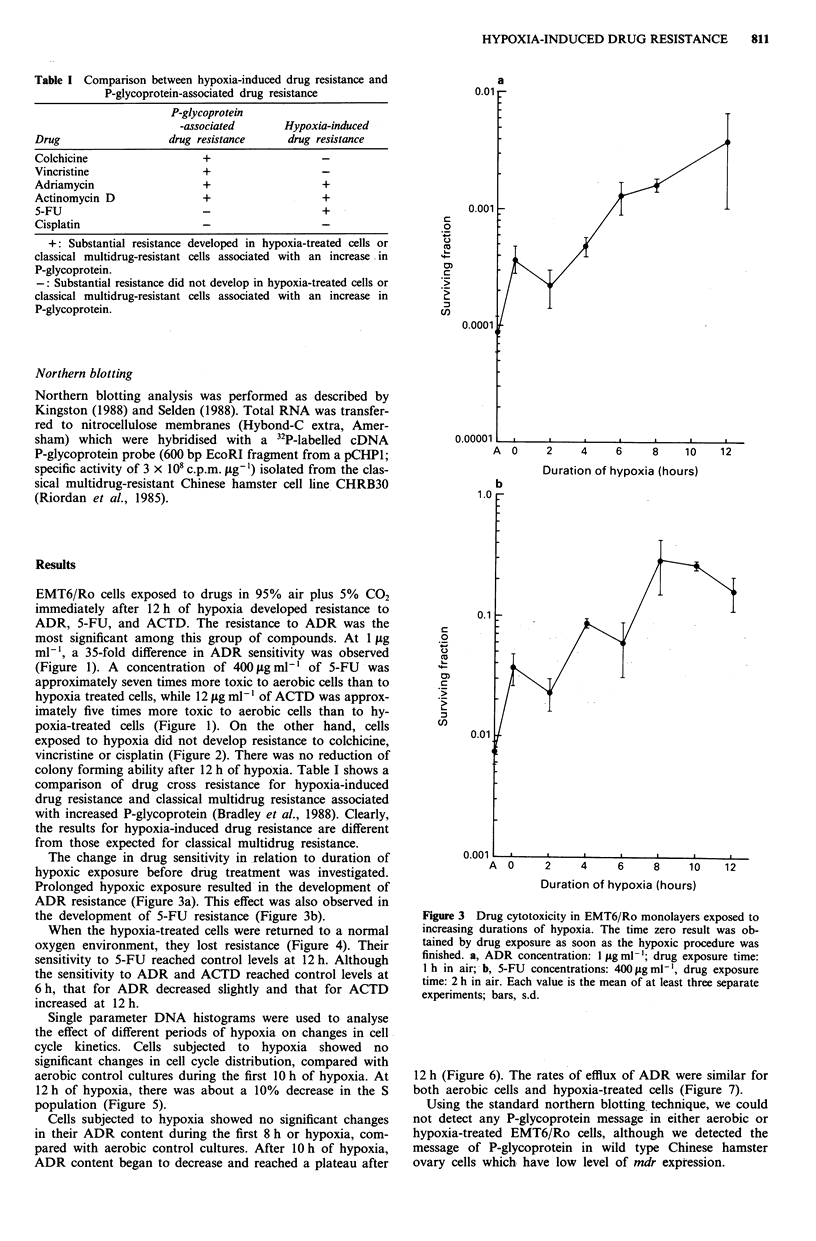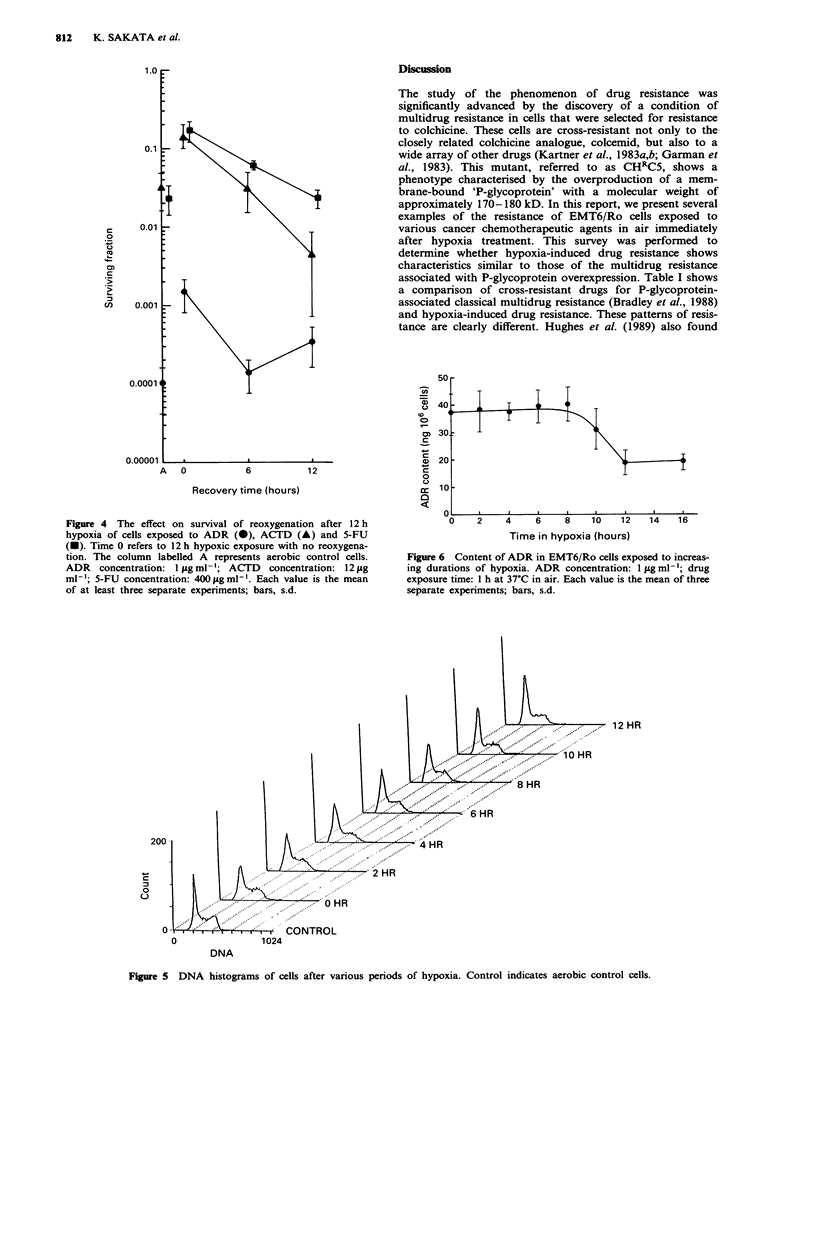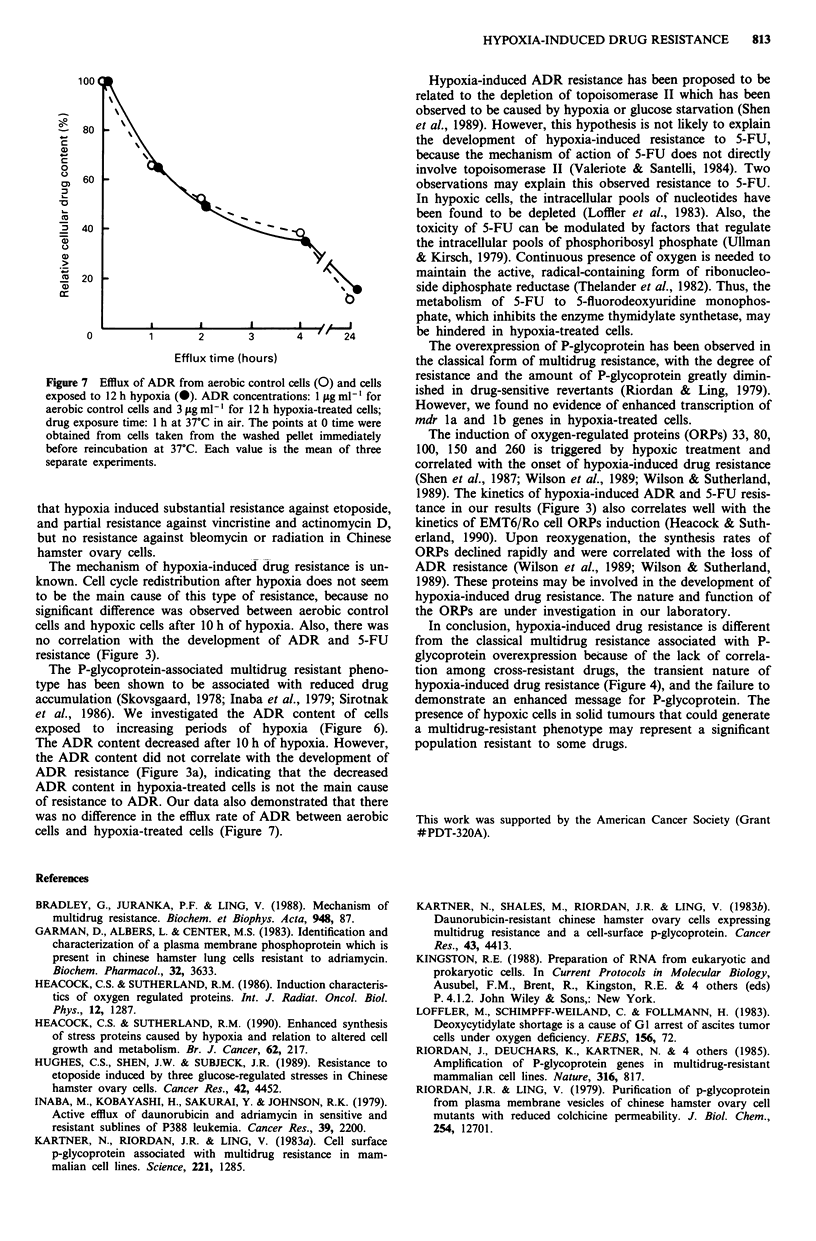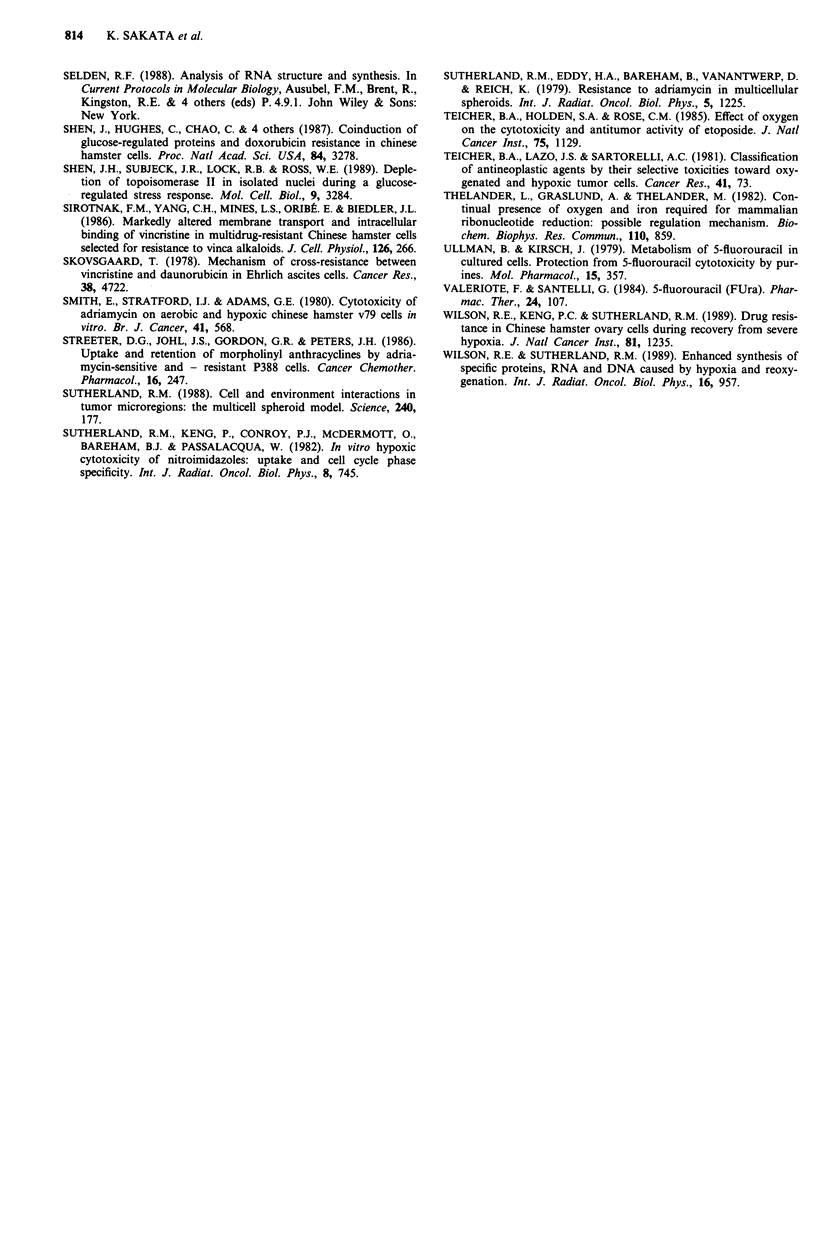# Hypoxia-induced drug resistance: comparison to P-glycoprotein-associated drug resistance.

**DOI:** 10.1038/bjc.1991.405

**Published:** 1991-11

**Authors:** K. Sakata, T. T. Kwok, B. J. Murphy, K. R. Laderoute, G. R. Gordon, R. M. Sutherland

**Affiliations:** Laboratory of Cell and Molecular Biology, Life Sciences Division SRI International, Menlo Park, California 94025.

## Abstract

In this report, we investigate several examples of hypoxia-induced drug resistance and compare them with P-glycoprotein associated multidrug resistance (MDR). EMT6/Ro cells exposed to drugs in air immediately after hypoxic treatment developed resistance to adriamycin, 5-fluorouracil, and actinomycin D. However, these cells did not develop resistance to colchicine, vincristine or cisplatin. When the cells were returned to a normal oxygen environment, they lost resistance. There was no correlation between the content of adriamycin and the development of adriamycin resistance induced by hypoxia. There was no difference between the efflux of adriamycin from aerobic cells and that from hypoxia-treated cells. The mRNA for P-glycoprotein was not detected in the hypoxia-treated cells. These results suggest that hypoxia-induced drug resistance is different from P-glycoprotein associated multidrug resistance.


Br. J. Cancer (1991), 64, 809-814 © Macmillan Press Ltd., 1991

### Hypoxia-induced drug resistance: comparison to P-glycoprotein-associated drug resistance

K. Sakata, T. Tak Kwok, B.J. Murphy, K.R. Laderoute, G.R. Gordon & R.M. Sutherland

*Laboratory of Cell and Molecular Biology, Life Sciences Division SRI International, Menlo Park, California 94025, USA.*

**Summary** In this report, we investigate several examples of hypoxia-induced drug resistance and compare them with P-glycoprotein associated multidrug resistance (MDR). EMT6/Ro cells exposed to drugs in air immediately after hypoxic treatment developed resistance to adriamycin, 5-fluorouracil, and actinomycin D. However, these cells did not develop resistance to colchicine, vincristine or cisplatin. When the cells were returned to a normal oxygen environment, they lost resistance. There was no correlation between the content of adriamycin and the development of adriamycin resistance induced by hypoxia. There was no difference between the efflux of adriamycin from aerobic cells and that from hypoxia-treated cells. The mRNA for P-glycoprotein was not detected in the hypoxia-treated cells. These results suggest that hypoxia-induced drug resistance is different from P-glycoprotein associated multidrug resistance.

As a tumour grows, heterogeneities of cellular microenvironments occur, such as the development of oxygen gradients in the tumour as a consequence of deficient vascularisation, and cause hypoxic cells that may be resistant to radiotherapy (Sutherland, 1988). Several studies using monolayer cultures (Smith *et al.*, 1980; Teicher *et al.*, 1981, 1985) and the multicell spheroid system (Sutherland *et al.*, 1979) have suggested that cells under hypoxic conditions or after hypoxic stress can be resistant to chemotherapy. The development of resistance to adriamycin and etoposide in Chinese hamster ovary cells after hypoxia has been shown to correlate with the induction of oxygen regulated proteins (ORPs) (Shen *et al.*, 1987; Hughes *et al.*, 1989; Wilson *et al.*, 1989). In this work, we investigate the effects of hypoxia on the development of resistance to various chemotherapeutic agents and perform experiments to investigate some possible mechanisms of these effects. Hypoxia-induced drug resistance is compared with P-glycoprotein associated multidrug resistance (MDR) (Bradley *et al.*, 1988).

### Materials and methods

### *Cell culture*

EMT6/Ro mammary tumour cells were maintained as exponentially growing monolayers in Basal Essential Eagle's growth media (Gibco) supplemented with 10% foetal bovine serum in humidified 5% $CO_2$/95% air, as described by Heacock and Sutherland (1986). All experiments were performed on monolayers prepared 24 h in advance by seeding $5 \times 10^5$ cells onto 60 mm glass petri dishes. Cells were 60 to 70% confluent at the time of the experiments.

### *Conditions of hypoxic exposure*

Before being gassed with $N_2$, the cells were supplied with 5 ml of fresh complete medium and allowed to equilibrate in a humidified 37°C incubator. Cells undergoing hypoxic stress were isolated in specially designed hypoxic chambers at room temperature (Sutherland *et al.*, 1982). The chambers were repeatedly evacuated and filled every 15 min for 2.25 h with the appropriate gas mixtures certified to contain less than 10 ppm $O_2$. The sealed chambers were then removed to a warm room (37°C) at a time point, t, referred to hereafter as '0' hours of hypoxia.

Correspondence: R.M. Sutherland.
Received 8 January 1991; and in revised form 20 May 1991.

### *Preparation of drugs and conditions of drug exposure*

All drugs used were obtained from Sigma Chemical Company. Stock solutions of adriamycin (ADR), vincristine, and actinomycin D (ACTD) were prepared with phosphate-buffered saline (PBS). Solutions of 5-fluorouracil (5-FU), colchicine, and cisplatin were made before each experiment. The solvents used were PBS for ADR, ACTD and cisplatin and distilled water for 5-FU. Absolute ethanol was used as a solvent for colchicine in order to obtain a sufficiently high concentration for the experiments. The final concentration of alcohol in the medium was 1%. As a control for the alcohol solvent, we established that 1% of absolute ethanol in cultures for 2 h had no effect on plating efficiency. Drug treatment was started under aerobic conditions at 37°C in the incubator after culture dishes were removed from the chambers at the zero time. Exposure times for ADR, ACTD and cisplatin were 1 h. A 2 h exposure time was used for 5-FU, colchicine and vincristine to cause significant cell killing. Exposure times were limited to 1-2 h to avoid the effect of release from a ORPs-induced state, which results in the loss of ADR resistance (Shen *et al.*, 1987; Wilson *et al.*, 1989). After treatment, the cells were harvested from monolayers with a 0.01% trypsin solution in citrate-buffered saline, counted, and plated for clonogenic cell survival assays. Each drug was tested in at least three separate experiments.

### *Analysis of cell cycle kinetics*

Cell cycle redistribution after various hypoxic exposures was examined by flow cytometric analysis of nuclei treated with propidium iodide (Sigma Chemical Company). Cells ($1 \times 10^6$) were trypsinised and fixed in 70% ethanol. After treatment with RNase at 1 mg $ml^{-1}$ for 30 min, the DNA was stained with propidium iodide at 10 $\mu g\ ml^{-1}$. Cells were analysed with an EPICS flow cytometer using laser excitation at 488 nm and a 600 nm wavelength-pass filter for propidium iodide fluorescence.

### *ADR content and efflux*

The ADR content was determined in cells treated the same way as those used for clonogenic cell survival. Cells were exposed to 1 $\mu g\ ml^{-1}$ of ADR for 1 h after various time periods of hypoxia, then trypsinised and pelleted at 4°C ($2 \times 10^6$ cells/pellet). Cell pellets were frozen at $-20$°C until analysed. For drug efflux determinations, aerobic control cells were exposed to 1 $\mu g\ ml^{-1}$ of ADR for 1 h while cells subjected to 12 h of hypoxia were exposed to 3 $\mu g\ ml^{-1}$ of ADR for 1 h, so that similar levels of ADR content could be obtained. The cells were washed three times with fresh

**Figure 1** The effect of hypoxia on drug toxicity. **a**, 1 h exposure to adriamycin; **b**, 1 h exposure to actinomycin D; **c**, 2 h exposure to 5-fluorouracil. Aerobic control cells (○); 12 h hypoxia-treated cells subsequently exposed to drugs in air (●). Each value is the mean of at least three separate experiments; bars, s.d.

**Figure 2** The effect of hypoxia on drug toxicity. **a**, 2 h exposure to colchicine; **b**, 2 h exposure to vincristine; **c**, 1 h exposure to cisplatin. Aerobic control cells (○); 12 h hypoxia-treated cells subsequently exposed to drugs in air (●). Each value is the mean of at least three separate experiments; bars, s.d.

medium, then reincubated. Extraction with 10 M urea was performed by solubilising the cell pellet in 0.2 ml of the reagent and incubating for 1 h at 37°C. After the incubation period, the urea solution was extracted twice with 0.5 ml of chloroform, the phases were separated by centrifugation at 12,000 *g* for 1 min and the chloroform extracts were combined and evaporated with nitrogen (Streeter *et al.*, 1986).

HPLC analysis was performed using a 4.6 × 150 mm column of Altex Ultrasphere octyl. The mobile phase was 70% methanol in 0.05 M phosphoric acid at a flow rate of 1.5 ml min$^{-1}$. Fluorometric detection was performed using an Aminco-Bowman spectrophotofluorometer at an excitation wavelength of 470 nm and an emission wavelength of 580 nm.

**Table I** Comparison between hypoxia-induced drug resistance and P-glycoprotein-associated drug resistance

| *Drug* | *P-glycoprotein -associated drug resistance* | *Hypoxia-induced drug resistance* |
|---|---|---|
| Colchicine | + | − |
| Vincristine | + | − |
| Adriamycin | + | + |
| Actinomycin D | + | + |
| 5-FU | − | + |
| Cisplatin | − | − |

+: Substantial resistance developed in hypoxia-treated cells or classical multidrug-resistant cells associated with an increase in P-glycoprotein.
−: Substantial resistance did not develop in hypoxia-treated cells or classical multidrug-resistant cells associated with an increase in P-glycoprotein.

*Northern blotting*

Northern blotting analysis was performed as described by Kingston (1988) and Selden (1988). Total RNA was transferred to nitrocellulose membranes (Hybond-C extra, Amersham) which were hybridised with a $^{32}$P-labelled cDNA P-glycoprotein probe (600 bp EcoRI fragment from a pCHP1; specific activity of $3 \times 10^8$ c.p.m. $\mu g^{-1}$) isolated from the classical multidrug-resistant Chinese hamster cell line CHRB30 (Riordan *et al.*, 1985).

### Results

EMT6/Ro cells exposed to drugs in 95% air plus 5% $CO_2$ immediately after 12 h of hypoxia developed resistance to ADR, 5-FU, and ACTD. The resistance to ADR was the most significant among this group of compounds. At 1 $\mu$g ml$^{-1}$, a 35-fold difference in ADR sensitivity was observed (Figure 1). A concentration of 400 $\mu$g ml$^{-1}$ of 5-FU was approximately seven times more toxic to aerobic cells than to hypoxia treated cells, while 12 $\mu$g ml$^{-1}$ of ACTD was approximately five times more toxic to aerobic cells than to hypoxia-treated cells (Figure 1). On the other hand, cells exposed to hypoxia did not develop resistance to colchicine, vincristine or cisplatin (Figure 2). There was no reduction of colony forming ability after 12 h of hypoxia. Table I shows a comparison of drug cross resistance for hypoxia-induced drug resistance and classical multidrug resistance associated with increased P-glycoprotein (Bradley *et al.*, 1988). Clearly, the results for hypoxia-induced drug resistance are different from those expected for classical multidrug resistance.

The change in drug sensitivity in relation to duration of hypoxic exposure before drug treatment was investigated. Prolonged hypoxic exposure resulted in the development of ADR resistance (Figure 3a). This effect was also observed in the development of 5-FU resistance (Figure 3b).

When the hypoxia-treated cells were returned to a normal oxygen environment, they lost resistance (Figure 4). Their sensitivity to 5-FU reached control levels at 12 h. Although the sensitivity to ADR and ACTD reached control levels at 6 h, that for ADR decreased slightly and that for ACTD increased at 12 h.

Single parameter DNA histograms were used to analyse the effect of different periods of hypoxia on changes in cell cycle kinetics. Cells subjected to hypoxia showed no significant changes in cell cycle distribution, compared with aerobic control cultures during the first 10 h of hypoxia. At 12 h of hypoxia, there was about a 10% decrease in the S population (Figure 5).

Cells subjected to hypoxia showed no significant changes in their ADR content during the first 8 h or hypoxia, compared with aerobic control cultures. After 10 h of hypoxia, ADR content began to decrease and reached a plateau after 12 h (Figure 6). The rates of efflux of ADR were similar for both aerobic cells and hypoxia-treated cells (Figure 7).

Using the standard northern blotting technique, we could not detect any P-glycoprotein message in either aerobic or hypoxia-treated EMT6/Ro cells, although we detected the message of P-glycoprotein in wild type Chinese hamster ovary cells which have low level of *mdr* expression.

**Figure 3** Drug cytotoxicity in EMT6/Ro monolayers exposed to increasing durations of hypoxia. The time zero result was obtained by drug exposure as soon as the hypoxic procedure was finished. **a**, ADR concentration: 1 $\mu$g ml$^{-1}$; drug exposure time: 1 h in air; **b**, 5-FU concentrations: 400 $\mu$g ml$^{-1}$, drug exposure time: 2 h in air. Each value is the mean of at least three separate experiments; bars, s.d.

**Figure 4** The effect on survival of reoxygenation after 12 h hypoxia of cells exposed to ADR (●), ACTD (▲) and 5-FU (■). Time 0 refers to 12 h hypoxic exposure with no reoxygenation. The column labelled A represents aerobic control cells. ADR concentration: 1 μg ml$^{-1}$; ACTD concentration: 12 μg ml$^{-1}$; 5-FU concentration: 400 μg ml$^{-1}$. Each value is the mean of at least three separate experiments; bars, s.d.

### Discussion

The study of the phenomenon of drug resistance was significantly advanced by the discovery of a condition of multidrug resistance in cells that were selected for resistance to colchicine. These cells are cross-resistant not only to the closely related colchicine analogue, colcemid, but also to a wide array of other drugs (Kartner et al., 1983a,b; Garman et al., 1983). This mutant, referred to as $CH^RC5$, shows a phenotype characterised by the overproduction of a membrane-bound 'P-glycoprotein' with a molecular weight of approximately 170–180 kD. In this report, we present several examples of the resistance of EMT6/Ro cells exposed to various cancer chemotherapeutic agents in air immediately after hypoxia treatment. This survey was performed to determine whether hypoxia-induced drug resistance shows characteristics similar to those of the multidrug resistance associated with P-glycoprotein overexpression. Table I shows a comparison of cross-resistant drugs for P-glycoprotein-associated classical multidrug resistance (Bradley et al., 1988) and hypoxia-induced drug resistance. These patterns of resistance are clearly different. Hughes et al. (1989) also found

**Figure 6** Content of ADR in EMT6/Ro cells exposed to increasing durations of hypoxia. ADR concentration: 1 μg ml$^{-1}$; drug exposure time: 1 h at 37°C in air. Each value is the mean of three separate experiments; bars, s.d.

**Figure 5** DNA histograms of cells after various periods of hypoxia. Control indicates aerobic control cells.

**Figure 7** Efflux of ADR from aerobic control cells (O) and cells exposed to 12 h hypoxia (●). ADR concentrations: 1 $\mu g\ ml^{-1}$ for aerobic control cells and 3 $\mu g\ ml^{-1}$ for 12 h hypoxia-treated cells; drug exposure time: 1 h at 37°C in air. The points at 0 time were obtained from cells taken from the washed pellet immediately before reincubation at 37°C. Each value is the mean of three separate experiments.

that hypoxia induced substantial resistance against etoposide, and partial resistance against vincristine and actinomycin D, but no resistance against bleomycin or radiation in Chinese hamster ovary cells.

The mechanism of hypoxia-induced drug resistance is unknown. Cell cycle redistribution after hypoxia does not seem to be the main cause of this type of resistance, because no significant difference was observed between aerobic control cells and hypoxic cells after 10 h of hypoxia. Also, there was no correlation with the development of ADR and 5-FU resistance (Figure 3).

The P-glycoprotein-associated multidrug resistant phenotype has been shown to be associated with reduced drug accumulation (Skovsgaard, 1978; Inaba *et al.*, 1979; Sirotnak *et al.*, 1986). We investigated the ADR content of cells exposed to increasing periods of hypoxia (Figure 6). The ADR content decreased after 10 h of hypoxia. However, the ADR content did not correlate with the development of ADR resistance (Figure 3a), indicating that the decreased ADR content in hypoxia-treated cells is not the main cause of resistance to ADR. Our data also demonstrated that there was no difference in the efflux rate of ADR between aerobic cells and hypoxia-treated cells (Figure 7).

Hypoxia-induced ADR resistance has been proposed to be related to the depletion of topoisomerase II which has been observed to be caused by hypoxia or glucose starvation (Shen *et al.*, 1989). However, this hypothesis is not likely to explain the development of hypoxia-induced resistance to 5-FU, because the mechanism of action of 5-FU does not directly involve topoisomerase II (Valeriote & Santelli, 1984). Two observations may explain this observed resistance to 5-FU. In hypoxic cells, the intracellular pools of nucleotides have been found to be depleted (Loffler *et al.*, 1983). Also, the toxicity of 5-FU can be modulated by factors that regulate the intracellular pools of phosphoribosyl phosphate (Ullman & Kirsch, 1979). Continuous presence of oxygen is needed to maintain the active, radical-containing form of ribonucleoside diphosphate reductase (Thelander *et al.*, 1982). Thus, the metabolism of 5-FU to 5-fluorodeoxyuridine monophosphate, which inhibits the enzyme thymidylate synthetase, may be hindered in hypoxia-treated cells.

The overexpression of P-glycoprotein has been observed in the classical form of multidrug resistance, with the degree of resistance and the amount of P-glycoprotein greatly diminished in drug-sensitive revertants (Riordan & Ling, 1979). However, we found no evidence of enhanced transcription of *mdr* 1a and 1b genes in hypoxia-treated cells.

The induction of oxygen-regulated proteins (ORPs) 33, 80, 100, 150 and 260 is triggered by hypoxic treatment and correlated with the onset of hypoxia-induced drug resistance (Shen *et al.*, 1987; Wilson *et al.*, 1989; Wilson & Sutherland, 1989). The kinetics of hypoxia-induced ADR and 5-FU resistance in our results (Figure 3) also correlates well with the kinetics of EMT6/Ro cell ORPs induction (Heacock & Sutherland, 1990). Upon reoxygenation, the synthesis rates of ORPs declined rapidly and were correlated with the loss of ADR resistance (Wilson *et al.*, 1989; Wilson & Sutherland, 1989). These proteins may be involved in the development of hypoxia-induced drug resistance. The nature and function of the ORPs are under investigation in our laboratory.

In conclusion, hypoxia-induced drug resistance is different from the classical multidrug resistance associated with P-glycoprotein overexpression because of the lack of correlation among cross-resistant drugs, the transient nature of hypoxia-induced drug resistance (Figure 4), and the failure to demonstrate an enhanced message for P-glycoprotein. The presence of hypoxic cells in solid tumours that could generate a multidrug-resistant phenotype may represent a significant population resistant to some drugs.

This work was supported by the American Cancer Society (Grant #PDT-320A).

### References

BRADLEY, G., JURANKA, P.F. & LING, V. (1988). Mechanism of multidrug resistance. *Biochem. et Biophys. Acta*, **948**, 87.

GARMAN, D., ALBERS, L. & CENTER, M.S. (1983). Identification and characterization of a plasma membrane phosphoprotein which is present in chinese hamster lung cells resistant to adriamycin. *Biochem. Pharmacol.*, **32**, 3633.

HEACOCK, C.S. & SUTHERLAND, R.M. (1986). Induction characteristics of oxygen regulated proteins. *Int. J. Radiat. Oncol. Biol. Phys.*, **12**, 1287.

HEACOCK, C.S. & SUTHERLAND, R.M. (1990). Enhanced synthesis of stress proteins caused by hypoxia and relation to altered cell growth and metabolism. *Br. J. Cancer*, **62**, 217.

HUGHES, C.S., SHEN, J.W. & SUBJECK, J.R. (1989). Resistance to etoposide induced by three glucose-regulated stresses in Chinese hamster ovary cells. *Cancer Res.*, **42**, 4452.

INABA, M., KOBAYASHI, H., SAKURAI, Y. & JOHNSON, R.K. (1979). Active efflux of daunorubicin and adriamycin in sensitive and resistant sublines of P388 leukemia. *Cancer Res.*, **39**, 2200.

KARTNER, N., RIORDAN, J.R. & LING, V. (1983*a*). Cell surface p-glycoprotein associated with multidrug resistance in mammalian cell lines. *Science*, **221**, 1285.

KARTNER, N., SHALES, M., RIORDAN, J.R. & LING, V. (1983*b*). Daunorubicin-resistant chinese hamster ovary cells expressing multidrug resistance and a cell-surface p-glycoprotein. *Cancer Res.*, **43**, 4413.

KINGSTON, R.E. (1988). Preparation of RNA from eukaryotic and prokaryotic cells. In *Current Protocols in Molecular Biology*, Ausubel, F.M., Brent, R., Kingston, R.E. & 4 others (eds) P. 4.1.2. John Wiley & Sons,: New York.

LOFFLER, M., SCHIMPFF-WEILAND, C. & FOLLMANN, H. (1983). Deoxycytidylate shortage is a cause of G1 arrest of ascites tumor cells under oxygen deficiency. *FEBS*, **156**, 72.

RIORDAN, J., DEUCHARS, K., KARTNER, N. & 4 others (1985). Amplification of P-glycoprotein genes in multidrug-resistant mammalian cell lines. *Nature*, **316**, 817.

RIORDAN, J.R. & LING, V. (1979). Purification of p-glycoprotein from plasma membrane vesicles of chinese hamster ovary cell mutants with reduced colchicine permeability. *J. Biol. Chem.*, **254**, 12701.

SELDEN, R.F. (1988). Analysis of RNA structure and synthesis. In *Current Protocols in Molecular Biology*, Ausubel, F.M., Brent, R., Kingston, R.E. & 4 others (eds) P. 4.9.1. John Wiley & Sons: New York.

SHEN, J., HUGHES, C., CHAO, C. & 4 others (1987). Coinduction of glucose-regulated proteins and doxorubicin resistance in chinese hamster cells. *Proc. Natl Acad. Sci. USA*, **84**, 3278.

SHEN, J.H., SUBJECK, J.R., LOCK, R.B. & ROSS, W.E. (1989). Depletion of topoisomerase II in isolated nuclei during a glucose-regulated stress response. *Mol. Cell. Biol.*, **9**, 3284.

SIROTNAK, F.M., YANG, C.H., MINES, L.S., ORIBÉ. E. & BIEDLER, J.L. (1986). Markedly altered membrane transport and intracellular binding of vincristine in multidrug-resistant Chinese hamster cells selected for resistance to vinca alkaloids. *J. Cell. Physiol.*, **126**, 266.

SKOVSGAARD, T. (1978). Mechanism of cross-resistance between vincristine and daunorubicin in Ehrlich ascites cells. *Cancer Res.*, **38**, 4722.

SMITH, E., STRATFORD, I.J. & ADAMS, G.E. (1980). Cytotoxicity of adriamycin on aerobic and hypoxic chinese hamster v79 cells *in vitro*. *Br. J. Cancer*, **41**, 568.

STREETER, D.G., JOHL, J.S., GORDON, G.R. & PETERS, J.H. (1986). Uptake and retention of morpholinyl anthracyclines by adriamycin-sensitive and - resistant P388 cells. *Cancer Chemother. Pharmacol.*, **16**, 247.

SUTHERLAND, R.M. (1988). Cell and environment interactions in tumor microregions: the multicell spheroid model. *Science*, **240**, 177.

SUTHERLAND, R.M., KENG, P., CONROY, P.J., MCDERMOTT, O., BAREHAM, B.J. & PASSALACQUA, W. (1982). *In vitro* hypoxic cytotoxicity of nitroimidazoles: uptake and cell cycle phase specificity. *Int. J. Radiat. Oncol. Biol. Phys.*, **8**, 745.

SUTHERLAND, R.M., EDDY, H.A., BAREHAM, B., VANANTWERP, D. & REICH, K. (1979). Resistance to adriamycin in multicellular spheroids. *Int. J. Radiat. Oncol. Biol. Phys.*, **5**, 1225.

TEICHER, B.A., HOLDEN, S.A. & ROSE, C.M. (1985). Effect of oxygen on the cytotoxicity and antitumor activity of etoposide. *J. Natl Cancer Inst.*, **75**, 1129.

TEICHER, B.A., LAZO, J.S. & SARTORELLI, A.C. (1981). Classification of antineoplastic agents by their selective toxicities toward oxygenated and hypoxic tumor cells. *Cancer Res.*, **41**, 73.

THELANDER, L., GRASLUND, A. & THELANDER, M. (1982). Continual presence of oxygen and iron required for mammalian ribonucleotide reduction: possible regulation mechanism. *Biochem. Biophys. Res. Commun.*, **110**, 859.

ULLMAN, B. & KIRSCH, J. (1979). Metabolism of 5-fluorouracil in cultured cells. Protection from 5-fluorouracil cytotoxicity by purines. *Mol. Pharmacol.*, **15**, 357.

VALERIOTE, F. & SANTELLI, G. (1984). 5-fluorouracil (FUra). *Pharmac. Ther.*, **24**, 107.

WILSON, R.E., KENG, P.C. & SUTHERLAND, R.M. (1989). Drug resistance in Chinese hamster ovary cells during recovery from severe hypoxia. *J. Natl Cancer Inst.*, **81**, 1235.

WILSON, R.E. & SUTHERLAND, R.M. (1989). Enhanced synthesis of specific proteins, RNA and DNA caused by hypoxia and reoxygenation. *Int. J. Radiat. Oncol. Biol. Phys.*, **16**, 957.